# Expanded functional roles of R2R3-MYB (S6) transcription factors in balancing phenylpropanoid and phenolamide pathways in Solanaceae

**DOI:** 10.1093/pcp/pcaf028

**Published:** 2025-03-13

**Authors:** Vincenzo D’Amelia, Anna Lisa Piccinelli, Teresa Docimo, Valerio Cirillo, Albino Maggio, Pasquale Chiaiese, Marco Possenti, Fabio D’Orso, Annalisa Staiti, Riccardo Aversano, Domenico Carputo

**Affiliations:** Department of Agricultural Sciences, University of Naples Federico II, Piazza Carlo di Borbone 1, Portici 80055, Italy; Department of Pharmacy, University of Salerno, Via Giovanni Paolo, II 132, Fisciano 84084, Italy; National Biodiversity Future Center (NBFC), Piazza Marina– Italia, Palermo 61 90133, Italy; Institute of Bioscience and BioResources, National Research Council, Via Università 100, Portici 80055, Italy; Department of Agricultural Sciences, University of Naples Federico II, Piazza Carlo di Borbone 1, Portici 80055, Italy; Department of Agricultural Sciences, University of Naples Federico II, Piazza Carlo di Borbone 1, Portici 80055, Italy; Department of Agricultural Sciences, University of Naples Federico II, Piazza Carlo di Borbone 1, Portici 80055, Italy; Council for Agricultural Research and Economics (CREA), Research Centre for Genomics and Bioinformatics, Via Ardeatina 546, Rome 00178, Italy; Council for Agricultural Research and Economics (CREA), Research Centre for Genomics and Bioinformatics, Via Ardeatina 546, Rome 00178, Italy; Department of Agricultural Sciences, University of Naples Federico II, Piazza Carlo di Borbone 1, Portici 80055, Italy; Department of Agricultural Sciences, University of Naples Federico II, Piazza Carlo di Borbone 1, Portici 80055, Italy; Department of Agricultural Sciences, University of Naples Federico II, Piazza Carlo di Borbone 1, Portici 80055, Italy

**Keywords:** gene duplication, hydroxycinnamoyl acid amides, paralogs, potato, *Solanum tuberosum*

## Abstract

Events of duplication and neo/subfunctionalization have significantly expanded the functional roles of R2R3 myeloblastosis (MYB) transcription factors in plants. In a previous study, we demonstrated that two paralogous R2R3 MYBs from *Solanum tuberosum* and *S. commersonii, AN1* and *AN2*, respectively, induce anthocyanin pigmentation to varying extents when transiently overexpressed. However, questions related to the distinct functions of these genes remained unanswered. In this study, we further investigated these genes by comparing transgenic tobacco plants that constitutively overexpress *AN1* and *AN2*. We observed differences between *AN1* and *AN2* that not only influenced plant pigmentation but also impacted the structural features of vascular tissues. Both genes promoted the accumulation of phenolamides; however, *AN1* showed a stronger capacity to regulate the phenylpropanoid pathway. In addition, our results suggest a potential role for *AN2* in regulating additional biological processes potentially involved in vascular development, as indicated by the GUS promoter localization study. Collectively, these results shed new light on the potentially ancestral functions of these R2R3 MYB genes, extending their known impact beyond anthocyanin biosynthesis.

## Introduction

Among the myriads of transcription factor (TF) families identified in plants, the myeloblastosis (MYB) stands out as versatile in orchestrating diverse physiological and developmental processes. MYB genes are ubiquitous across eukaryotes, suggesting an ancient origin (Dubos et al. 2010, De Mendoza et al. 2013, Du et al. 2015). MYBs diversify according to the region responsible for recognizing and binding specific DNA sequences. Indeed, MYB proteins are categorized into different classes based on the number and the identity of adjacent repeats (R1, R2, R3, and R4) comprising the MYB binding domain, with the R2R3 MYBs being the most prevalent in plant genomes (Martin and Paz-Ares 1997, Riechmann and Ratcliffe 2000, Dubos et al. 2010). R2R3 MYB proteins have evolved extensively and exhibit differential patterns of diversification across various plant groups, including angiosperms and nonflowering plants like mosses, liverworts, and lycophytes (Du et al. 2015, Jiang and Rao 2020, Wu et al. 2022). Gene duplication followed by subfunctionalization or neofunctionalization has expanded their functional roles within specific lineage and species (Jiang and Rao 2020, Wu et al. 2022), with gene counts ranging from 68 in *Ginko biloba* to 244 in *Glycine max* (Du et al. 2012, Yang et al. 2021).

R2R3 MYBs play key roles in regulating both primary and secondary metabolism, determining cell fate, controlling developmental processes, and responding to environmental stressors (Feller et al. 2011, Chang et al. 2020, Jiang and Rao 2020, Wu et al. 2022). Originally, 25 subgroups (here defined as subfamilies) of the R2R3-MYB gene family were identified in *Arabidopsis thaliana* (Stracke et al. 2001). They have expanded to 90 as more species- or lineage-specific subfamilies have been considered (Du et al. 2015, Jiang and Rao 2020). R2R3 MYB proteins belonging to the same subfamily usually share similar functions. Subfamily 6 (S6), for instance, regulates anthocyanin biosynthesis in plants (Dubos et al. 2010). This is a well-studied function of R2R3 MYB that involves interaction within the MYB–bHLH–WD40 (MBW) activator complex, which also includes basic helix-loop-helix (bHLH) and WD40repeat TFs (Koes et al. 2005, Ramsay and Glover 2005, Montefiori et al. 2015). MYBs from other subfamilies or even other families (e.g. R3 MYB) can interfere with the MBW complex to repress the anthocyanin production in a negative feedback fashion (Albert et al. 2014, LaFountain and Yuan 2021).

A plethora of research articles have identified anthocyanin activators mainly within the S6 family (see reviews by Lin-Wang, et al. 2010, Liu, et al. 2015, Liu et al. 2018, Naing and Kim 2018). This prevalence can be attributed to the distinct phenotype they induce—a visible purple pigmentation—when overexpressed in experimental settings. Anthocyanins, responsible for the red, blue, and purple hues in plants, are synthesized from common phenylpropanoid precursors (e.g. coumarate, naringenin, and dihydrokaempferol) by enzymes encoded by several structural genes. An intriguing aspect of R2R3 MYB regulators is that homologs do not always regulate the same subset of structural genes, as evidenced by pioneering studies that compared petunia, maize, and snapdragon (Martin and Paz-Ares 1997, Jin and Martin 1999). This variability also extends to paralogous R2R3 MYBs; despite their strong similarity in DNA-binding domains, they display flexibility in target recognition and functional divergence (Jin and Martin 1999, Zhou et al. 2018, Ma et al. 2024). The most apparent effect triggered by R2R3 MYBs in S6 is a visible accumulation of anthocyanins. This prominent impact on the phenotype has mainly attracted scientific interest, thereby limiting the exploration of R2R3 MYBs’ potential pleiotropic effects. The persistence of a sort of ancestral function in these duplicated genes may provide significant insights into the functional evolution of R2R3 MYBs and their role in environmental adaptation. In this framework, some studies showed that the divergence and pleiotropic functions of MYBs can be highlighted and studied through ectopic expression in heterologous systems (Outchkourov et al. 2014, Wen and Chu 2017, D’Amelia et al. 2018, Ma et al. 2024).

In a previous investigation, we studied R2R3-MYBs in potato, focusing on *StAN1* and *StAN2* genes within the Developer (D) locus of *Solanum tuberosum*, along with their homologs *ScAN1* and *ScAN2* isolated from the wild potato *S. commersonii* (D’Amelia et al. 2018). Structural differences were observed between *StAN1* and *ScAN1*, even though both robustly activate anthocyanin production when transiently overexpressed in *Nicotiana benthamiana*. In contrast, the sequences of *StAN2* and *ScAN2* were remarkably conserved, suggesting that *St/ScAN2* is the ancestral gene that was later duplicated to evolve into AN1. Overexpression of St/ScAN2 in *N. benthamiana* led to low levels of anthocyanins, but induced a senescent phenotype, indicating a possible subfunctionalization event.

In the current study, we investigated pleiotropic effects and structural divergence between *AN1* and *AN2* genes beyond anthocyanin production. We compared the phenotyes resulting from the overexpression in *N. tabacum* of *ScAN2*, isolated from *S. commersonii,* and *StAN1,* isolated from a purple potato (*S. tuberosum*) variety. Our results revealed the activation of distinctive metabolic pathways and potential structural tissue modifications. Histological analyses of tobacco transgenic plants and GUS tissue localization studies in potato further validated the functional diversity of *StAN1* and *ScAN2* proteins. These observations suggest an ecological role for gene duplication in the Solanaceae family.

## Results

### ScAN2 and StAN1 have distinct metabolic influence

**Figure 1.**
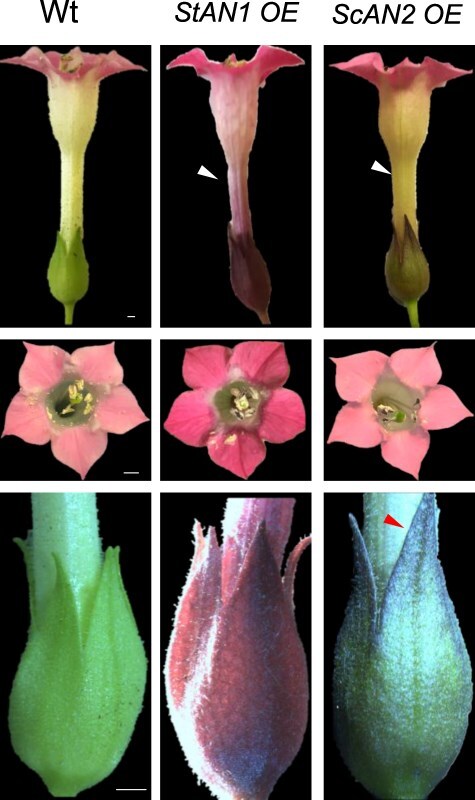
Different pigmentation patterns in tobacco flowers are induced by p35S:*StAN1* and p35S:ScAN2. Pigmentation phenotypes in stable transgenic *StAN1* overexpressing (*StAN1* OE) and *ScAN2* overexpressing (*ScAN2* OE) lines compared to Wt tobacco cv. Samsun. The top panel: young open flowers; middle panel: corollas; bottom panel: sepals. An arrow highlights differences in pigmentation. Scale bar = 1 mm.

**Figure 2.**
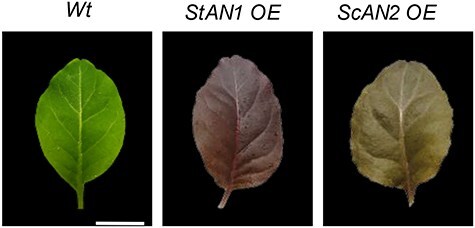
Different pigmentation patterns in tobacco leaves are induced by p35S:*StAN1* and p35S:*ScAN2*. Pigmentation phenotypes of stable transgenic tobacco *StAN1* overexpressing (*StAN1* OE) and *ScAN2* overexpressing (*ScAN2* OE) lines compared to Wt tobacco cv. Samsun. Scale bar = 2 cm.

*Nicotiana tabacum* cv Samsun lacks evident anthocyanin accumulation in vegetative tissues, and its flowers are pigmented only in the lobes (Figs 1 and 2). Representative T3 plants that overexpress (OE) either *StAN1* or *ScAN2* under 35S (CaMV35S) promoter (p35S) displayed diverse anthocyanin pigmentation across all tissues (Figs. 1 and 2). Flowers of *StAN1* OE lines showed enhanced petal blush and an entire tube pigmentation, traits absent in both Wt and *ScAN2* OE lines. Likewise, the sepals of *StAN1* OE lines displayed uniform pigmentation, whereas in ScAN2 OE lines only the edges exhibited purple pigmentation (Fig. 1). These phenotypic traits remained consistent across independent transgenic lines and clonal copies. The superior ability of *StAN1* compared to *ScAN2* in activating anthocyanin production when constitutively expressed was further qualitatively evidenced by different amounts of anthocyanins accumulated in the leaves of the two transgenic tobacco lines (Fig. 2; **Table 1**). Total anthocyanin content was lower in young leaves of *ScAN2* OE than in *StAN1* OE plants (Supplementary Fig. S1).

**Table 1.** Putatively annotated and identified discriminant metabolites of *StAN1* and *ScAN2* OE transgenic tobacco lines

			Ratio			Log2 Area fold change			*q* ^c^		
Compounds ^a^	IL ^b^	RSD QC	*StAN1* OE1/Wt	*ScAN2* OE/Wt	*StAN1* OE/*ScAN2* OE	*StAN1* OE1/Wt	ScAN2 OE/Wt	StAN1OE/ScAN2 OE	*StAN1* OE1/Wt	ScAN2 OE/Wt	StAN1OE /ScAN2 OE
Anthocyanins											
Cyanidin 3-*O*-Hex-dHex	2	1	707.5	204.4	3.5	9.5	7.7	1.8	<.001	.003	<.001
Cyanidin 3-*O*-Hex	2	2	109.0	43.6	2.5	6.8	5.5	1.3	<.001	.008	.001
Dihydrochalcone/flavanones/dihydroflavonols/flavonols											
Taxifolin *O*-Hex	2	2	140.9	35.5	4.0	7.1	5.2	2.0	<.001	.041	<.001
Eriodictyol *O*-Hex	2	3	120.7	3.9	30.6	6.9	2.0	4.9	<.001	*1.000*	<.001
Taxifolin	1	5	64.7	21.3	3.0	6.0	4.4	1.6	<.001	.004	<.001
Phloretin	2	3	61.6	33.8	1.8	5.9	5.1	0.9	<.001	<.001	.002
Dihydroisorhamnetin	3	5	59.6	17.8	3.4	5.9	4.2	1.7	<.001	.002	<.001
Dihydrorobinetin *O*-Hex-dHex	2	15	49.9	13.8	3.6	5.6	3.8	1.9	<.001	.037	<.001
Quercetin *O*-Hex-dHex-Hex	2	3	30.1	31.6	1.0	4.9	5.0	−0.1	<.001	<.001	1.000
Isorhamnetin 3-*O*-Hex-dHex	2	3	21.9	17.3	1.3	4.5	4.1	0.3	<.001	<.001	.003
Quercetin-*O*-Hex	2	5	7.9	2.8	2.8	3.0	1.5	1.5	<.001	*.054*	<.001
Rutin	1	1	5.2	3.8	1.4	2.4	1.9	0.4	<.001	<.001	.008
HCA derivatives											
Dicaffeoylquinic acid-Hex	2	4	68.1	33.8	2.0	6.1	5.1	1.0	<.001	.001	.001
*p*-Coumaroylquinic acid	2	2	8.5	0.9	9.3	3.1	−0.1	3.2	<.001	*1.000*	<.001
HCA amides (phenolamides)											
*N*1,*N*3-Dicaffeoylspermidine	2	2	427.3	326.8	1.3	8.7	8.4	0.4	<.001	.002	1.000
*N*-Caffeoylputrescine-Hex	3	2	226.0	153.2	1.5	7.8	7.3	0.6	.007	.026	1.000
*N*-Caffeoylspermidine isomer 3	2	2	196.2	153.8	1.3	7.6	7.3	0.4	<.001	.002	1.000
*N*-*p*-Coumaroylputrescine	2	2	193.3	308.2	0.6	7.6	8.3	−0.7	.050	.002	.270
*N*-Caffeoylputrescine	2	2	174.3	211.8	0.8	7.5	7.7	−0.3	.001	<.001	.265
*N*-Caffeoylspermidine-Hex	3	11	125.6	44.4	2.8	7.0	5.5	1.5	.001	*.250*	.215
*N*-Feruloylspermidine	2	1	77.4	53.3	1.5	6.3	5.7	0.5	<.001	.007	1.000
*N*-*p*-Coumaroylspermidine	2	3	68.7	71.7	1.0	6.1	6.2	−0.1	.008	.005	1.000
*N*-Caffeoylputrescine-Hex-dHex	3	3	42.0	70.0	0.6	5.4	6.1	−0.7	.024	.002	.506
*N*-Hydroxycaffeoylspermidine	3	4	31.6	19.8	1.6	5.0	4.3	0.7	<.001	.002	.244
*N*-Caffeoylspermidine isomer 2	2	2	30.9	21.3	1.5	5.0	4.4	0.5	.001	.008	1.000
*N*1-Caffeoyl-*N*3-dihydrocaffeoylspermidine	2	1	18.2	30.6	0.6	4.2	4.9	−0.7	<.001	<.001	.003
*N*-Feruloylputrescine	2	4	12.3	12.4	1.0	3.6	3.6	0.0	.003	.001	.914
*N*-Dihydrocaffeoylspermidine	3	4	8.7	14.8	0.6	3.1	3.9	−0.8	<.001	<.001	.010
*N*-Caffeoylspermidine isomer 1	2	8	2.1	4.5	0.5	1.1	2.2	−1.1	.311	.001	.022

a Hex: hexoside, dHex: deoxyhexoside.

b Identification levels according to MSI.

c *q*: adjusted *P*-value using Benjamini–Hochberg false discovery rate of 5% (nonsignificant values in italics).

A high-performance liquid chromatography coupled to high-resolution mass spectrometry (UHPLC–HRMS/MS)-untargeted metabolomic analysis was performed to evaluate the changes in secondary metabolite profiles in leaves of *ScAN2* and *StAN1* OE lines. Identified (29 out 70, Supplementary Table S1) secondary metabolites differentially abundant in ScAN2 OE and StAN1 OE compared to the wild type (Wt) (*P* < .05 and log2 fold > 2) were regrouped in four different classes (**Table 1**). The accumulation patterns of anthocyanins, particularly cyanidin mono- and di-hexoside, differed substantially between the lines (*q* < .001 StAN1 OE/ScAN2 OE; **Table 1**). While the patterns of flavonoids and hydroxycinnamic acids (HCAs) induced by the two R2R3 MYB constructs were similar, the overexpression of *StAN1* more effectively induced the accumulation of these molecules compared to *ScAN2* (**Table 1**). Key examples include the glucoside form of eriodyctiol and *p*-coumaroylquinic acid, which exhibited ∼4.9- and 3.2-fold more induction in *StAN1* OE than *ScAN2* OE lines. Besides phenylpropanoid molecules, we detected in both transgenic lines a significant increase in abundance (Log2 fold change 3.1–8.7; *q* < .05 StAN1 OE/Wt and ScAN2 OE/Wt) of hydroxycinnamoyl acid amides (HCCAs), also known as phenolamides. The main molecules detected were the conjugation of coumaroyl, feruloyl, and caffeoyl groups with spermidine and putrescine with a prevalence of spermidine conjugates (**Table 1**). Unlike flavonoids and HCA, the abundance of most HCCAs was not significantly different between *StAN1* OE and *ScAN2* OE lines (*q* > .05), although specific molecules like *N*1-caffeoyl-*N*3-dihydrocaffeoylspermidine and *N*-dihydrocaffeoylspermidine (*q* = .01) were slightly more accumulated in *ScAN2* OE plants.

### *ScAN2* OE and *StAN1* OE tobacco plants differ transcriptionally

**Figure 3.**
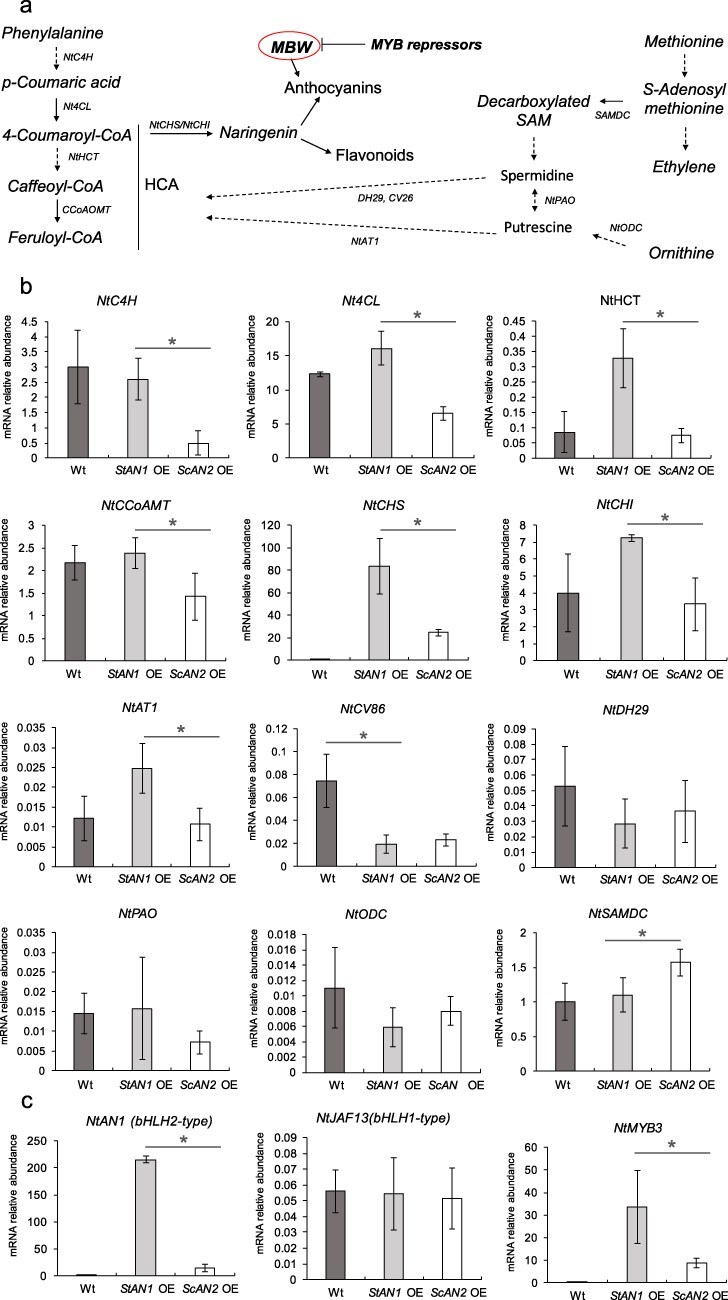
*ScAN2* is a weak activator of the phenylpropanoid pathway and bHLH cofactors. (a) Biochemical routes leading to anthocyanin and HCA production, including acetylation with polyamines to produce HCCAs (phenolamides). (b) Relative mRNA expression levels of structural genes in the phenylpropanoid and phenolamide pathways in Wt, *StAN1* overexpressing (*StAN1* OE), and *ScAN2* overexpressing (*ScAN2* OE) tobacco lines. (c) Relative mRNA expression levels of anthocyanin regulatory genes in Wt, *StAN1* OE, and *ScAN2* OE. Values are means ± SD (*n* = 3). Asterisks indicate statistically significant differences (*P < 0.05, Student’s *t*-test).

The expression of the main structural genes of the HCA and flavonoid pathways confirmed the lower ability of *ScAN2* to trigger these genes compared to *StAN1* when overexpressed in tobacco plants (Fig. 3a–c). Indeed, the transcripts of the key genes *Nt4CL* and *NtHCT* were significantly higher in fully expanded leaves of *StAN1* OE lines compared to *ScAN2* OE lines (Fig. 3b). Unexpectedly, the transcript levels in *ScAN2* OE were even lower than those in Wt. Conversely, the expression of *NtCHS*, which catalyzes the phenylpropanoid route to flavonoid biosynthesis, was higher in *ScAN2* OE leaves than the Wt, but still 4-fold lower than in plants overexpressing AN1 (Fig. 3b). We also investigated transcripts of genes involved in phenolamide formation. Three genes (*NtAT1, NtCV86*, and *NtDH29*) encoding for enzymes involved in the conjugation of polyamines with HCA (*N*-hydroxycinnamoyltransferase activity) were monitored (Fig. 3b). Of these, only *NtAT1*, involved in the conjugation of HCAs with putrescine (AT1), was notably up-regulated in *StAN1* OE. Additionally, the expression of the S-adenosylmethionine decarboxylase (SAMD)-encoding gene, key in the initial step of the polyamine biosynthesis, was ∼1.5-fold higher in *ScAN2* OE than *StAN1* OE or Wt.

Overexpression of our R2R3-MYBs also triggered the expression of TFs within the MYB–bHLH complex that regulates flavonoid biosynthesis (Fig. 3a). Hence, we monitored the expression of three endogenous tobacco bHLH cofactors. *NtAN1* (bHLH-type2) expression was 8-fold higher in *StAN1* OE plants compared to *ScAN2* OE (Fig. 3a and c). *NtJAF13* (bHLH-type1) displayed similar transcript levels among the three lines (Fig. 3c). The third cofactor, *NtMYB3* (S4), is an R2R3-MYB protein that contains an ethylene-responsive element binding factor–associated amphiphilic repression (EAR) motif. The *Arabidopsis* ortholog was previously shown to inhibit anthocyanins in tobacco (Dubos et al. 2010, Zhou et al. 2017). Consistent with feedback mechanisms in which the activator complex triggers the expression of repressors (Alberts et al. 2014), *NtMYB3* transcripts were upregulated in both transgenic plants, but 4-fold more in *StAN1* OE lines.

### Constitutive overexpression of *ScAN2* and *StAN1* induces structural morphological changes in tobacco

**Figure 4.**
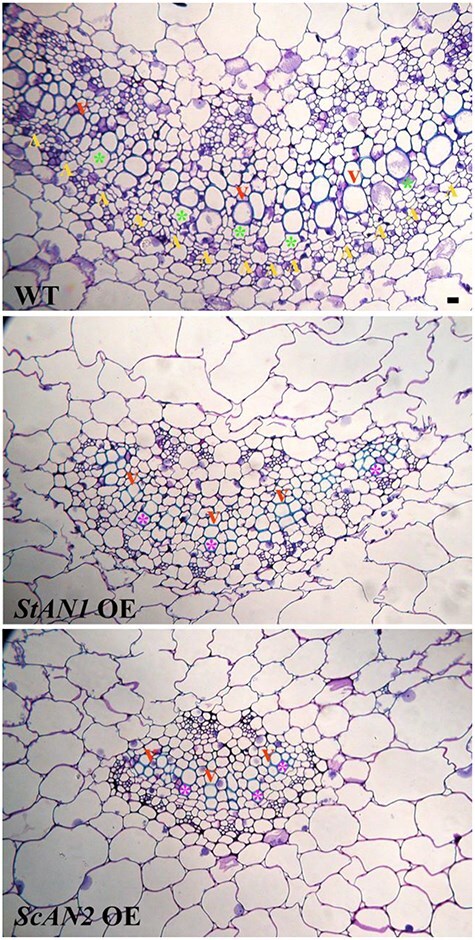
The overexpression of *StAN1* and *ScAN2* produced structural changes. Midrib cross-sections of *N. tabacum* Wt leaves, *StAN1* overexpressing (*StAN1* OE), and *ScAN2* overexpressing (*ScAN2* OE) tobacco lines. Different development of the vein is shown with a strong reduction in the vein caliber and xylem cell lumens. Furthermore, lines over-expressing these genes show a reduction in cambium continuity and its proliferative activity. Annotations: proliferating cambium cells (yellow arrowhead), differentiated metaxylem cells (red arrowhead), differentiating metaxylem cells in contact with the cambium (green asterisk in Wt), and differentiated metaxylem cells in contact with the cambium (pink asterisk in StAN1 OE and ScAN2 OE). Scale bars: 20 μm.

From the comparison of the central veins of Wt leaves with those in *ScAN1* OE and *ScAN2* OE (Fig. 4), it was evident that the ectopic and constitutive expression of these genes reduced the size of the vascular bundle. Figure 4 also shows that the effect of the constitutive expression of both *ScAN1* and *ScAN2* extends beyond the vascular bundle size. In leaves of plants constitutively expressing *ScAN2*, there was a significant reduction in cambial extension and activity. In fact, while in the Wt the cambial arc was almost continuous along the entire vascular bundle, it was fragmented in both *StAN1* OE and *ScAN2* OE lines. This was particularly evident in *ScAN2* OE. In both *StAN1* OE and *ScAN2* OE lines, the metaxylem exhibited a significant reduction in cell lumen compared to the Wt. Additionally, in *ScAN2* OE plants, the mature metaxylem (lignified wall) directly abutted the cambium, while in the Wt a differentiating metaxylem cell separated the two regions.

**Figure 5.**
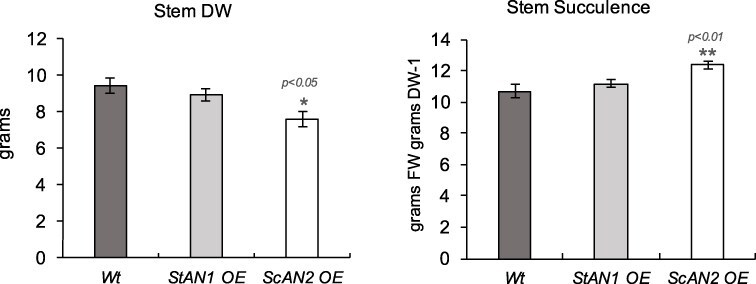
Biometric differences in Wt tobacco plants compared to *StAN1* overexpressing (*StAN1* OE) and *ScAN2* overexpressing (*ScAN2* OE) lines. Dry weight (DW) and succulence in the stems StAN1 OE, ScAN2 OE, and Wt plants. Asterisks indicate statistically significant differences (*P-value according to Student’s *t*-test).

Compared to the Wt, the leaves of *StAN1* OE line accumulated less dry biomass, whereas the leaves of *ScAN2* OE line showed no change (Supplementary Fig. S2). Regarding the stem, *ScAN2* OE lines displayed a significant reduction in dry matter compared to the Wt (Fig. 5). This reduction correlated with the lower xylem differentiation identified by the anatomical analysis of the central vein, thus providing further evidence of the altered cambial activity induced by *ScAN2* OE. Additionally, there was a general reduction in lignin content in young leaves of *ScAN2* OE lines (Supplementary Fig. S3). The tissues in the StAN1 OE leaves and *ScAN2* OE stems showed significantly higher succulence than the Wt, indicating a lower dry matter accumulation (Supplementary Figs S2 and S5).

### *ScAN2* promoter activity localized also in acyanic tissues

**Figure 6.**
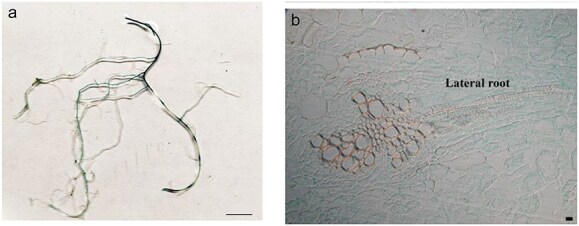
ScAN2 promoter activity localizes also in acyanic tissues. (a) Macroscopic localization of GUS activity in potato roots under the control of pAN2 (≈1700 bp) promoter. (b) Cross-section of the main potato root above the second-order lateral root insertion. Key structures are labeled: Cortex (pink asterisk), endodermis (orange asterisk), pericycle (yellow asterisk), phloem pole (P), and xylem pole (X). Scale: 1 cm for (a) and 10 μm for (b).

The previous findings suggested an ancient role of anthocyanin regulatory MYBs in driving the production of additional compounds that impact plant development. Being interested in understanding the ancestral role of these genes rather than their specialization, we decided to focus on AN2 by investigating its spatial expression. We characterized tobacco and potato plants transformed with ≈1700 bp of the *ScAN2* 5ʹ upstream sequence fused to the *GUS* reporter gene (pAN2::GUS). Results showed a leaf and root localization pattern in both plant systems (Fig. 6; Supplementary Figs. S4–S6). In tobacco, there was also localization in the upper part of the buds where anthocyanins typically accumulate (Fig. 1; Supplementary Fig. S6B). We also characterized *S. tuberosum* cv Desiree: *in vitro* leaflets showed GUS expression mostly in leaf veins and, randomly, in mesophyll areas (Supplementary Fig. S4). We also examined the non-anthocyanin (acyanic) root tissue of potato plants. One month from micro-propagation, when roots were fully developed, GUS activity was highly detectable throughout the main roots and fully expanded secondary roots (Fig. 6). When the differentiation zone was almost complete, GUS staining was highly localized at the junction with lateral roots (Fig. 6a, Supplementary Fig. S5). Cross-sections of these junctions revealed GUS presence in the vascular stele from the pericycle onwards and in the cortical layers (Fig. 6b).

## Discussion

Within the angiosperm, the R2R3 MYB gene family continues to evolve, marking divergences in lineage adaptation. In the genus *Solanum*, for example, a specific duplication of the R2R3 MYB S6 occurred, leading to duplicated genes at the potato locus D and the tomato locus Aft (Jung et al. 2009, Powel et al. 2022). Our findings on *StAN1* and *ScAN2* fit with the ongoing evolution of the R2R3 MYB family. We investigated the functional differences between these two R2R3 MYB genes, examining their roles in secondary metabolism and their impact on plant development.

### Variation in the quantity and localization of anthocyanins in *N. tabacum*

*StAN1* was more effective in promoting anthocyanin accumulation compared to *ScAN2* confirming findings by D’Amelia et al. (2018). Compared to our previous work, the experiments reported here delve deeper into the distinct contributions of these two TFs in regulating phenylpropanoids toward anthocyanin production. *StAN1* is finely tuned toward anthocyanins and, more broadly, flavonoids. This specificity results from its close association with the MBW complex, essential for activating anthocyanin biosynthesis. This complex includes the R2R3 MYB TF S6, two bHLHs from different clades of group IIIf, and WD40 TFs. In tobacco, it has been established that the R2R3 MYB anthocyanin activator induces the expression of *NtAN1* (bHLH type 2), according to a hierarchical transcriptional regulatory cascade (Albert et al. 2014). Our data indicate that *StAN1* (the R2R3 MYB) is more adept at activating the expression of *NtAN1* (bHLH type 1) compared to *ScAN2*, underscoring its different molecular abilities in directing the phenylpropanoid pathway toward anthocyanins. Thole and colleagues (2019) discussed the distinct capabilities of R2R3 MYBs from various berry species to activate anthocyanin biosynthesis in tobacco due to their varying interaction with endogenous bHLH partners. We concur and suggest that these differences may also involve the different capabilities to trigger the transcripts of bHLH type 1 (*NtAN1*) before interacting with the proteins. Further investigations are needed to determine whether these effects result from an interaction between the MYBs and NtJAF13 (bHLH type 2). As proposed by Montefiori et al. (2015), in Solanaceae PhJAF13 is required to activate or enhance the expression of genes encoding bHLH type 1 proteins. Consequently, the nature of interaction between MYBs and JAF13 can impact the whole activation of anthocyanin biosynthesis.

The lower ability of *ScAN2* to form MBW complexes may also be suggested by the lower transcript level of the anthocyanin repressor MYB3 (belonging to S4 and containing the EAR motif) detected in our *ScAN2* OE lines. Likely, NtMYB3 works similarly to MYB27 in Petunia (S4), where its expression, activated by the MBW complex, modulates anthocyanin production in a negative feedback fashion (Albert et al. 2014). It is well known that the phenylpropanoid pathway gene expression correlates with precursors available through different feedback mechanisms (Reuben et al. 2013). This would explain why the expression of some genes in the phenylpropanoid pathway was downregulated in *ScAN2* OE lines compared to the Wt. This can be a consequence of an unbalanced ability of *ScAN2* to activate the structural genes. Another interesting aspect is that the lower specialization of *ScAN2* toward anthocyanin biosynthesis is visually apparent due to the lack of pigmentation in flower tubes and sepals of transgenic tobacco plants (Fig. 1). Being both TFs under the same constitutive promoter, the different tissue pigmentations observed may result from a negative post-transcriptional/translational regulation. The floral tissues of our plants could provide valuable material for future analyses aimed at elucidating the distinct molecular functional abilities of these proteins beyond binding dynamics.

### *StAN1* and *ScAN2* activate phenolamides and differentially influence developmental traits

*ScAN2*, unlike *Sc/StAN1*, has a limited capacity to induce molecular complexes involved in anthocyanin biosynthesis and precursors. However, it effectively activates the production of phenolamides, likely due to its influence on the expression of genes involved in the primary polyamine pathway (i.e. *NtSAMDC*). In our previous work, we observed that the expression of ScAN2 in the wild potato *S. commersonii* was significantly activated endogenously by cold stress; in addition, this activation was associated with phenolamide accumulation in its leaves (D’Amelia et al. 2018). Notably, *StAN1* also retains the ability to induce phenolamide accumulation, despite its subfunctionalization toward stronger anthocyanin biosynthesis. In Solanaceous plants, R2R3-MYB TFs not only regulate the phenylpropanoid metabolism but also control the primary precursors from the shikimate pathway and aromatic amino acid biosynthesis (Zhang et al. 2015, Martin et al. 2023). Consistently, our tobacco plants overexpressing *StAN1* showed a 47% increase in total leaf amino acid content compared to the Wt, with a significant rise in ornithine levels. This is in line with the presence of these alkaloid-derived compounds reported here. This suggests that the varying degrees of subfunctionalization in MYB genes have redirected the metabolic pathways from primary compounds to more specialized metabolites with specific functions. Hence, we decided to understand if this wide metabolic perturbation could also correspond to structural differences with a possible physiological impact. Our results provide evidence that overexpression of either *StAN1* or *ScAN2* affects the development of vascular bundles. Reuben et al. (2013) and Gaquerel et al. (2013) demonstrated that, in genetically modified *Arabidopsis*, phenolamides participate in lignin biosynthesis. This is consistent with the reduced lignin but increased phenolamides accumulation in tobacco *StAN1* OE and *ScAN2* OE lines. It was also relevant to observe that the overexpression of *ScAN2* and *StAN1* impacted the development of vascular bundles with varying degrees of effect. The reduction of vascular bundles in young leaves was more pronounced in *ScAN2* OE, which is in line with their significantly lower lignin levels. Evidence also suggests that the two genes have different effects on plant development. Indeed, compared to Wt *StAN1*, OE plants show reduced dry biomass at the leaf level, while those overexpressing *ScAN2* exhibit similar reductions at the stem level. Dry matter partitioning among plant organs may overall affect plant water relations. *StAN1* has been previously shown to confer higher drought tolerance by inducing morpho-anatomical leaf changes that lead to reduced plant water use (Cirillo et al. 2021). Exploring whether a similar drought tolerance could be induced by increased stem succulence in *ScAN2* OE lines may provide insights into how water distribution in different plant organs affects drought tolerance. This possible adaptive mechanism has been recently investigated in *Zea mays* (Yan et al. 2023).

### Evolutionary significance of anthocyanin/phenolamide coregulation

Based on the above discussion, we propose that S6 R2R3 MYB could have an impact in coregulating, either directly or indirectly, the production of phenolamides besides anthocyanin/flavonoids. This is a shared ability of *StAN1* OE and *ScAN2* OE plants, albeit with small differences. Solanaceus have shown a propensity to coregulate these metabolic pathways throughout their evolutionary history. In fact, there are MYBs specialized in regulating phenolamides, which have retained a partial ability to activate the phenylpropanoid pathway by sharing common gene targets (Onkokesung et al. 2012, Yogendra et al. 2017). This is the case of tobacco MYB8 from *Nicotiana attenuate*, which is specifically involved in phenolamide production and also contributes to lignin accumulation by targeting hydroxycinnamoyl-CoA:shikimate/quinate hydroxycinnamoyl transferase (HCT) (Onkokesung et al. 2012). It is similar to *StMYB8* isolated from potato, which activates genes involved in lignin (*HCT*, *PHT*, and *CHS*) and flavonoid biosynthesis (*F3H*) (Yogendra et al. 2017). However, *MYB8-like* directly activates genes encoding *N*-hydroxycinnamoyltransferases, key in catalyzing the polyamine– HCA conjugation for forming phenolamides (Kaur et al. 2010, Onkokesung et al. 2012). Our transcriptional data indicated that neither *ScAN2* nor *StAN1* induces the expression of *NtAT1, NtDH2*, and *NtCV86*, suggesting that the formation of hydroxycinnamoyl amides in our transgenic lines may result from basal acetyltransferases activity and an increased precursor abundance; alternatively, different HXXXD-type acyl-transferase might be involved. By contrast, MYB8 seems to have evolved a specific ability to conjugate polyamine with HCA by using *NtAT1, NtDH2*, and *NtCV86*. In a similar study in tomato, Cao et al. (2024) recently identified another MYB, SlMYB13, which is directly involved in activating a cluster of HXXXD-type acyl-transferase genes, leading to phenolamide accumulation. This functional redundancy among certain MYB proteins in regulating phenolamide production further supports our hypothesis that some of these proteins may have evolved new functions while retaining some ancestral capabilities.

Since the ability to activate HCA along with phenolamides has been fully retained by paralogs, i.e. *ScAN2* and *StAN1* along with other R2R3 MYBs (including MYB8 or MYB13), it is plausible to hypothesize that it corresponds to an ancestral function. A recent and accurate phylogenetic analysis of R2R3 MYBs performed by Jiang and Rao (2020) reorganized the relative position of R2R3 MYB, considering deeper evolutionary aspects. They classified anthocyanin genes previously identified by Dubos et al. (2010) in the S6 group, along with genes closely related to MYB8-like, within the superfamily VIII. The authors suggested that genes of this superfamily have undergone multiple duplications, with some R2R3-MYB genes retaining partial ancestral functions. We agree with Jiang and Rao (2020) that changes in expression patterns and functional diversification are a way of differentiating duplicated genes during evolution and we also add that the R2R3 MYB represents an exemplary case. The localization of ScAN2 promoter activity within root inner tissues supports its ancestral function beyond anthocyanins synthesis. The ability to drive GUS expression in vascular tissues of both roots and leaves was already observed for R2R3 MYBs of the S6 clade. Interestingly, this localization has been previously documented during plant senescence (Warner 2008). This observation is intriguing and warrants further investigation, as there are indications that R2R3 MYBs belonging to the S6 clade could be involved in senescence mechanisms, as reported previously in tobacco (D’Amelia et al. 2018) and *Liquidambar formosana* (Wen and Chu 2017).

Our findings support the idea that environmental pressures may have maintained a shared regulation between phenolamides and flavonoids within the Solanaceae lineage (Onkokesung et al. 2012, Yogendra et al. 2017, Li et al. 2020). We propose two main, yet not alternative, hypotheses within a general ecological perspective. First, anthocyanins and phenolamides may synergistically act as defensive compounds, potentially modulating ROS and senescent effects. This was evidenced by their simultaneous accumulation in tobacco plants silenced for the strigolactone pathway, which indicates a role in herbivore defense (Li et al. 2020). Second, the copresence of anthocyanins may mitigate the physiological negative effects of nitrogen pool deprivation caused by phenolamide production during such a biotic attack. Previous studies have shown that phenolamide production reallocates nitrogen from growth patterns to defense mechanisms, thus reducing photosynthesis (Ullmann‐Zeunert et al. 2013, He et al. 2022). In such a situation, anthocyanins, due to their biochemical and light absorbing proprieties, may help to buffer the excess light energy generated by a photosynthesis reduction. This concept can be also applied in the context of abiotic stress. Our results from drought stress experiments on *StAN1* OE tobacco plants (Cirillo et al. 2021) suggested that anthocyanins may help plants adapt to stressful conditions by reducing photosynthesis through the absorption of certain wavelengths of the solar spectrum.

In conclusion, R2R3 MYB TFs are recognized for their ability to control multiple plant processes, affecting both specialized and primary metabolism. Several indications lead to the hypothesis that the S6 family, known to be specifically involved in anthocyanin production, can also link C- and N-derived pathways in Solanaceae through phenolamides. From our results, we conclude that the ability to trigger phenolamide accumulation is an ancestrally retained capacity, stressed through the genetic overexpression of these genes. Whether this is a direct or indirect regulation is still to be discovered and potential novel HXXXD-type acyl-transferase that can be the target of our genes can be discovered through combined –omic approaches. However, at this stage of investigation, we underline that there is an evolutionary advantage to genetically inter-regulate anthocyanin and phenolamide pathways. The pleiotropic effect of these genes can impact developmental traits and give a physiological advantage during biotic or abiotic stress. While we acknowledge that direct mutagenesis may allow for a more precise delineation of the distinct roles of the two TFs, the current data from ectopic and constitutive expression experiments have already provided valuable insights. These findings reveal striking differences between *StAN1* and *ScAN2* and demonstrate how the balance between two classes of compounds can influence various developmental and ecological aspects in plants.

## Materials and Methods

### Plant materials and growth conditions

Transgenic tobacco (*N. tabacum* cv. Samsun) plants carrying either p35S:*StAN1* produced in D’Amelia et al. (2014) or p35S:*ScAN2* (this study) were used. Wt plants were also included as control. Seeds of all plants (T2) used were germinated separately in styrofoam trays. One month after germination, 10 plants per genotype with similar levels of transgene expression were transplanted in 21-cm-diameter pots under greenhouse conditions with the controlled temperature set at 28°C. Two months after transplanting, three biological representative plants of T3 were chosen from the different lines and used as biological replicates. These have been used for metabolomic, molecular, and microscopy analyses.

The tetraploid (2*n* = 4*x* = 48) potato (*S. tuberosum*) commercial variety Désireé was also used in this study. Plants were propagated *in vitro* on Murashige and Skoog (MS) medium (Sigma-Aldrich, http://www.sigmaaldrich.com) with 1% (w/v) sucrose and 0.8% (w/v) agar and incubated at 24°C, exposed to an irradiance of 200 μmol m^−2^ s^−1^ and under a 16-h/8-h (light/dark) photoperiod before transformation procedure.

### Plasmid material and construction

The plasmid containing p35S:*ScAN2*, under the regulation p35S CaMV, was obtained as described in D’Amelia et al. (2018). The 5ʹ upstream sequence of *ScAN2* (from −1 to −1740 nts from the ATG) was isolated from the gDNA of *S. commersonii* (PI243503). Primers for the amplification of the 5ʹ upstream region of ScAN2 were designed based on the sequence information from scaffold4043 of the *Solanum commersonii* genome (Aversano et al. 2015). Primers were designed with flanking attB sites to perform gateway cloning (i.e. using BP and LR recombinase ) following manufacturer’s instruction (Gateway Cloning Protocols, Thermo Fisher Scientific, www.thermofisher.com). In particular, the following primers were used: Fw: **GGGGACAAGTTTGTACAAAAAAGCAGGCT**TCCGTGTATTTGTTTTCGATAATGG; Rv: **GGGGACCACTTTGTACAAGAAAGCTGGGTC**TTTTTTTTAATAATATATAGTTCTAAG. The fragment was recombined in attL sites of pMDC164 in the upstream region to the GUS sequence. The obtained pAN2::GUS vector was checked through Sanger sequencing.

### Tobacco and potato transformation

*Agrobacterium tumefaciens* strain LBA4404 was used for genetic transformation. Transformation of p35S::AN2 and pAN2::GUS was carried out by cocultivation of *N. tabacum* cv Samsun leaf explants with *A. tumefaciens* in accordance with (Horsch et al. 1985). pAN2::GUS was also transformed in *S. tuberosum* cv Désirée according to Visser et al. (1991). Leaf explants were excised from sterile plants grown *in vitro*. Shoots regenerated on selective regeneration medium by using hygromycin 30 mg l^−1^ for potato and hygromycin 50 mg l^−1^ for tobacco in selective media. Briefly, for potato *A. tumefaciens*-mediated transformation was performed by growing cultures in LB medium with selective antibiotics, followed by inoculation of precultured explants in diluted MS10 medium. Explants were transferred to M400 medium (MS10; 2 mg l^−1^ zeatin, 0.01 mg l^−1^ NAA, 0.1 mg l^−1^ GA_3_, and 30 mg l^−1^ hygromycin) containing cefotaxime. Green calluses were isolated and transferred to M13 medium (MS30; 0.25 mg l^−1^ BA, 0.1 mg l^−1^ GA_3_, and 30 mg l^−1^ hygromycin) for shoot induction, and independent shoots were selected and rooted on MS30 medium. Successfully rooted shoots were used for GUS staining.

### RNA extraction and qRT-PCR

Total RNA was extracted from leaves of tobacco plants using the Spectrum Plant Total RNA Kit (Sigma-Aldrich) and following the manufacturer’s guidelines. One microgram of total RNA was reverse transcribed to cDNA using an oligo-dT(20) primer and SuperScript III reverse transcriptase (Invitrogen, www.invitrogen.com) in a 20-μl final reaction. Quantitative RT-PCR was used to monitor the expression of target genes, employing primers previously reported by D’Amelia et al. (2018). New primers for structural genes involved in phenolamide biosynthesis are listed in Supplementary Table S2. The experiment was carried out in biological triplicates using the 2× QuantiFast SYBR Green PCR Master Mix (Qiagen, www.qiagen.com) and the ABI PRISM 7900HT Instrument (www.thermofisher.com).

### Metabolomic analyses

Dried leaf samples (50 ± 2 mg) were used with 2 ml of methanol:H_2_O:formic acid (80:19:1 v/v) by ultrasound-assisted solid–liquid extraction for 30 min at 25°C in a thermostat-controlled ultrasound bath. The supernatants were pooled after centrifugation and evaporated to dryness using a SpeedVac. The extracts were reconstituted with 5 ml of methanol:H_2_O:formic acid (20:79:1 v/v) and filtered through 0.20 µM regenerated cellulose filters before the chromatographic analysis. Quality control (QC) samples were prepared by mixing equal volumes of all sample extracts.

UHPLC–HRMS/MS analyses were carried out using an UltiMate 3000 UHPLC system interfaced to a Q-Exactive mass spectrometer (ThermoFisher Scientific, Milano, Italy), equipped with a heated electrospray ionization source (HESI-II). A Kinetex C18 column (2.1 × 100 mm, 1.6 μm; Phenomenex, Bologna, Italy) and a binary gradient (0–2 min, 2% B; 2–8 min, 2%–10% B; 8–15 min, 10%–25% B; 15–18 min, 25%–60% B; 18–19 min, 60%–98% B; 19–23 min, 98% B) of H_2_O (A) and acetonitrile (B), both containing 1% of HCOOH, at 30°C and a flow rate of 500 µl min^−1^, were employed for the chromatographic separations. MS detection was performed in positive ionization mode and using a full MS data-dependent MS/MS acquisition mode. The resolution of full MS (range 100–1000 m/z) and dd-MS2 scans were set at 70k and 17.5k (FWHM), respectively. A normalized collision energy between 30 and 50 was applied. Instrument control and spectra acquisition were carried out using Xcalibur software (Version 4.4, ThermoFisher Scientific).

UHPLC–HRMS raw data files (samples, procedural blank, and QC) were processed by Compound Discoverer software (v. 3.3, Thermo Fisher Scientific) using a predefined untargeted metabolomics processing workflow with statistics to detect unknown compounds and to perform the differential analysis. Briefly, the study used categorical factors in the form and the ratios StAN1/Wt, ScAN2/Wt, and StAN1/ScAN2 were defined for the differential analysis. Workflow parameters were set as follows: peak alignment, ±5 ppm and ±0.4 min; compound detection, ±5 ppm, ±0.2 min, S/N ratio >3, peak intensity >500 000, isotope intensity tolerance <30%; and peak integration, normalized peak areas to the total area of the corresponding samples. Results were then filtered for background subtraction (procedural blank), retention time range (>1 min and <17 min), peak area (>1 000 000), relative standard deviation (RSD) of the QCs (% RSD < 30%), and elimination of in-source fragments. For each compound, group area ratio and fold change (expressed as log2 scale) were calculated and the variations were evaluated using both a one-way ANOVA model with Tukey as *post hoc* test (*P*-value) and a Benjamini–Hochberg correction for the false-discovery rate (adjusted *P*-value, termed *q* value).

The discriminant metabolites (*P* < 0.05 and log2 fold > 2) were annotated from the Volcano plots following data filtering. The identity of these metabolites was verified based on HRMS, MS/MS spectra, and RT. The presumed identity was confirmed by comparison with the reference standards (Level 1 of Metabolomics Standards Initiative, MSI) when available, or putatively assigned based on HRMS/MS spectra reported in literature data and databases (MSI, Level 2). When no spectrum or literature information was available, the detected unknown was tentatively assigned based on the interpretation of the HRMS/MS fragmentation pattern (MSI, Level 3).

### UV-visible spectrometric investigation

Total monomeric anthocyanin content was estimated using the pH differential spectrum method of Giusti and Wrolstad (2001) with freeze-dried samples. Lignin content was estimated on fresh samples from three plants per genotype by performing acetyl bromide lignin (ABSL) extraction according to Yokoyama et al. (2002) and Moreira-Vilar et al. (2014), with slight modifications reported in Cirillo et al. (2021).

### GUS-staining and microscopy analyses

The second pair of *StAN1* OE and *ScAN2* OE tobacco leaves (the basal part of the leaf) were collected and fixed in ethanol 70%. Samples were dehydrated through a graded series of ethanol and embedded in Technovit 7100 (Kulzer, Hereaus, Wehreim, Germany) as indicated by the manufacturer. These tissues were stained with 0.1% toluidine blue. Transgenic materials carrying pAN2::GUS were used for GUS staining. In particular, shoots, primary roots, and flowers of tobacco plants along with leaflets and roots of 1-month-old potato micro-propagated plants were rinsed with water and immersed in fixation solution (10 mM MES, pH 5.6, with 300 mM mannitol and 0.3% formaldehyde). Staining was performed using β-Glucuronidase Reporter Gene Staining Kit following the manufacturer’s instructions (Sigma-Aldrich Co.). For microscopic observation, transverse sections of samples were taken using a Microm HM 325 microtome (Microm, Walldorf, Germany). Sections of leaves were stained. Sections were viewed with an Axioskop 2 Plus (Zeiss, Germany) binocular microscope equipped with DIC optics. Images were taken with the Coolpix 990 (Nikon, Japan).

## Supplementary Material

pcaf028_Supp

## Data Availability

Untargeted UHPLC–HRMS metabolite profiles of StAN1 and ScAN2 OE transgenic tobacco lines are available in the Zenodo repository database (DOI 10.5281/zenodo.13982987) .
