# Supplementary material for: Expanded functional roles of R2R3-MYB (S6) transcription factors in balancing phenylpropanoid and phenolamide pathways in Solanaceae
Source: Plant Cell Physiol. 2025 Mar 13;66(6):878–89. doi: 10.1093/pcp/pcaf028 (PMC12290281; doi:10.1093/pcp/pcaf028)
Supplement: pcaf028_Supp [file pcaf028_supp.zip › suppl_data/pcp-2024-e-00254-File009.docx]

**Table S1.** UHPLC-HRMS/MS data of discriminant metabolites of StAN1 and ScAN2 OE transgenic tobacco lines.

| **N** | **Compound** | **RT (min)** | **Molecular formula** | **[M+H]^+^ (m/z)** | **Error (ppm)** | **Diagnostic product ions**  **(m/z)** | **MSI level ^b^** |
| --- | --- | --- | --- | --- | --- | --- | --- |
| 2 | N-dihydrocaffeoylspermidine | 1.02 | 310.2124 | C_16_H_27_N_3_O_3_ | -0.6 | 293.18570, 286.93271, 258.93774, 239.13855, 222.11237, 165.05440, 123.04406 | 3 |
| 3 | unknown | 1.02 | 373.0441 | - | - | - | - |
| 4 | N-caffeoylspermidine is1 | 1.18 | 308.1967 | C_16_H_25_N_3_O_3_ | -0.7 | 291.17014, 234.11212, 220.09656, 163.03877, 146.16512, 145.02827, 135.04402, 129.13866, 112.11221, 89.10764 | 2 |
| 5 | unknown | 1.40 | 185.0445 | - | - | - | - |
| 6 | unknown | 1.41 | 415.0720 | - | - | - | - |
| 7 | unknown | 1.41 | 139.0389 | - | - | - | - |
| 8 | unknown | 1.41 | 431.0497 | - | - | - | - |
| 9 | N-hydroxycaffeoylspermidine | 1.59 | 324.1916 | C_16_H_25_N_3_O_4_ | -0.5 | 307.16495, 250.10703, 236.09152, 179.03372, 161.02312, 146.16499, 133.02838, 129.13860, 112.11223, 89.10740 | 3 |
| 10 | unknown | 1.88 | 385.0441 | - | - | - | - |
| 11 | unknown | 1.88 | 369.0666 | - | - | - | - |
| 12 | unknown | 1.92 | 401.0391 | - | - | - | - |
| 13 | unknown | 1.92 | 385.0614 | - | - | - | - |
| 14 | unknown | 2.07 | 253.1546 | - | - | - | - |
| 15 | N-caffeoylspermidine is2 | 2.23 | 308.1967 | C_16_H_25_N_3_O_3_ | -0.5 | 291.17001, 237.12325, 220.09656, 163.03873, 146.16496, 145.02829, 135.04381, 129.13852, 112.11209 | 2 |
| 16 | unknown | 2.75 | 207.5391 | - | - | - | - |
| 17 | unknown | 2.75 | 385.0614 | - | - | - | - |
| 18 | unknown | 2.75 | 339.0686 | - | - | - | - |
| 19 | unknown | 2.75 | 401.0390 | - | - | - | - |
| 20 | unknown | 2.76 | 341.0447 | - | - | - | - |
| 21 | N-caffeoylspermidine is3 | 2.78 | 308.1966 | C_16_H_25_N_3_O_3_ | -0.9 | 291.17001, 234.11234, 220.09683, 163.03882, 146.16508, 145.02832, 135.04398, 112.11221, 89.10762 | 2 |
| 22 | N-caffeoylspermidine-Hex | 2.96 | 470.2497 | C_22_H_35_N_3_O_8_ | 0.0 | 308.19629, 291.17053, 234.11230, 220.09723, 163.03876, 145.02809, 135.04364, 129.13860, 115.12302, 98.09666, | 3 |
| 23 | unknown | 3.13 | 339.0476 | - | - | - | - |
| 24 | unknown | 3.14 | 323.0737 | - | - | - | - |
| 25 | unknown | 3.14 | 385.0443 | - | - | - | - |
| 26 | unknown | 3.92 | 415.0548 | - | - | - | - |
| 27 | N-p-coumaroylspermidine | 4.59 | 292.2018 | C_16_H_25_N_3_O_2_ | -0.6 | 275.17502, 218.11758, 204.10188, 147.04385, 146.16516, 119.04919, 112.11217 | 2 |
| 28 | N-caffeoylputrescine | 5.20 | 251.1386 | C_13_H_18_N_2_O_3_ | -1.9 | 234.11224, 163.03877, 135.04405, 89.10768 | 2 |
| 29 | unknown | 5.20 | 249.1235 | - | - | - | - |
| 30 | unknown | 5.21 | 401.0390 | - | - | - | - |
| 31 | unknown | 5.22 | 155.0338 | - | - | - | - |
| 32 | N-caffeoylputrescine-Hex | 5.48 | 413.1918 | C_19_H_28_N_2_O_8_ | -0.2 | 329.14911, 251.13835, 234.11212, 163.03874, 135.04396, 89.10765 | 3 |
| 33 | N-feruloylspermidine | 6.36 | 322.2124 | C_17_H_27_N_3_O_3_ | -0.3 | 305.18625, 248.12790, 234.11232, 177.05447, 146.16501, 145.02827 | 2 |
| 34 | unknown | 6.45 | 410.2173 | - | - | - | - |
| 35 | unknown | 6.52 | 184.0387 | - | - | - | - |
| 36 | unknown | 6.52 | 367.0699 | - | - | - | - |
| 37 | N-p-coumaroylputrescine | 6.72 | 235.1441 | C_13_H_18_N_2_O_2_ | 0.1 | 218.11720, 147.04378, 119.04910, 89.10760 | 2 |
| 38 | unknown | 6.72 | 383.0648 | - | - | - | - |
| 39 | N-caffeoylputrescine-Hex-dHex | 6.78 | 559.2498 | C_25_H_38_N_2_O_12_ | 0.1 | 359.15958, 329.14908, 251.13852, 234.11255, 163.03876, 135.04384, 89.10770 | 3 |
| 40 | unknown | 6.86 | 426.2122 | - | - | - | - |
| 41 | unknown | 7.28 | 427.0549 | - | - | - | - |
| 42 | taxifolin O-hexose | 7.31 | 467.1185 | C_21_H_22_O_12_ | 0.3 | 305.0655, 287.05499, 259.05997, 231.06508, 195.02892, 167.0339, 153.01814, 149.02325, 123.04408 | 2 |
| 43 | Dicaffeoylquinic acid-Hex | 7.65 | 517.1553 | C_22_H_28_O_14_ | 0.6 | 163.03868, 135.04387 | 2 |
| 44 | N-feruloylputrescine | 8.15 | 265.1545 | C_14_H_20_N_2_O_3_ | -0.6 | 248.12808, 177.05437, 163.03862, 149.05952, 145.02820, 89.10767 | 2 |
| 45 | unknown | 8.15 | 381.1152 | - | - | - | - |
| 46 | unknown | 8.47 | 349.1257 | - | - | - | - |
| 47 | unknown | 8.47 | 365.0996 | - | - | - | - |
| 48 | unknown | 8.82 | 427.0911 | - | - | - | - |
| 49 | unknown | 8.82 | 365.1206 | - | - | - | - |
| 50 | unknown | 8.82 | 381.0947 | - | - | - | - |
| 51 | Dihydroisorhamnetin (methyltaxifolin) | 9.40 | 319.0811 | C_16_H_14_O_7_ | -0.4 | 301.07028, 273.07639, 245.08063, 195.02910, 167.03377, 153.01811, 149.02322, 137.05965 | 3 |
| 52 | Dihydrorobinetin O-Hex-dHex | 9.60 | 613.1765 | C_27_H_32_O_16_ | 0.3 | 305.06570, 287.05505, 179.03357, 137.02322 | 2 |
| 53 | unknown | 9.66 | 305.1018 | - | - | - | - |
| 54 | QuercetinO-Hex-dHex-hex | 9.85 | 773.2135 | C_33_H_40_O_21_ | 0.1 | 465.16364, 303.0495, 153.01839 | 2 |
| 55 | p-coumaroylquinic acid | 10.01 | 339.1074 | C_16_H_18_O_8_ | -0.2 | 165.05453, 147.04382, 119.04917 | 2 |
| 56 | unknown | 10.01 | 254.0336 | - | - | - | - |
| 57 | unknown | 10.01 | 259.0125 | - | - | - | - |
| 58 | Cyanidin 3-O-Hex (kuromanin) | 10.22 | 449.1078 ^c^ | C_21_H_21_O_11_ | -0.1 | 287.05484, 137.02327 | 1 |
| 59 | Cyanidin 3-O-Hex-dHex (keracyanin) | 10.70 | 595.1652 ^c^ | C_27_H_31_O_15_ | -1.0 | 287.05444, 137.02330 | 2 |
| 60 | Taxifolin | 10.76 | 305.0654 | C_15_H_12_O_7_ | -0.7 | 287.05579, 259.05981, 231.06502, 195.02893, 167.03377, 153.01810, 149.02321, 137.02325, 123.04403 | 1 |
| 61 | N1-caffeoyl-N3-dihydrocaffeoylspermidine | 11.15 | 472.2441 | C_25_H_33_N_3_O_6_ | -0.3 | 455.21875, 310.21216, 308.19629, 165.05452, 163.03879, 135.04416, 123.04408 | 2 |
| 62 | unknown | 11.44 | 264.0179 | - | - | - | - |
| 63 | unknown | 11.44 | 343.0424 | - | - | - | - |
| 64 | N1,N3-dicaffeoylspermidine | 11.81 | 470.2285 | C_25_H_31_N_3_O_6_ | -0.2 | 308.19653, 234.11226, 220.09656, 163.03874, 146.16483, 145.02824,135.04396 | 2 |
| 65 | Eriodictyol O-Hex | 12.40 | 451.1234 | C_21_H_22_O_11_ | -0.1 | 289.07025, 179.03371, 163.03868, 153.01797, 135.04382 | 2 |
| 66 | Quercetin O-Hex | 12.78 | 465.1028 | C_21_H_20_O_12_ | 0.1 | 303.04962, 153.01814 | 2 |
| 67 | Rutin | 12.99 | 611.1603 | C_27_H_30_O_16_ | -0.5 | 303.04965, 153.01842 | 1 |
| 68 | Isorhamnetin 3-O-Hex-dHex | 14.16 | 625.1764 | C_28_H_32_O_16_ | 0.2 | 317.06512, 302.04166, 153.01801 | 2 |
| 69 | Phloretin | 14.66 | 275.0914 | C_15_H_14_O_5_ | -0.1 | 169.04941, 107.04932 | 2 |
| 70 | unknown | 18.36 | 381.2424 | - | - | - | - |

^a^ Hex: hexoside, dHex: deoxyhexoside; ^b^ Identification levels according to Metabolomics Standards Initiative; ^c^ corresponging to [M]^+^ ion.
