# Supplementary material for: Expanded functional roles of R2R3-MYB (S6) transcription factors in balancing phenylpropanoid and phenolamide pathways in Solanaceae
Source: Plant Cell Physiol. 2025 Mar 13;66(6):878–89. doi: 10.1093/pcp/pcaf028 (PMC12290281; doi:10.1093/pcp/pcaf028)
Supplement: pcaf028_Supp [file pcaf028_supp.zip › suppl_data/pcp-2024-e-00254-File010.docx]

Table S2. Primers used in this study

| ***Primer*** | ***Sequence 5'->3'*** | | ***Target gene/Reference sequence*** |
| --- | --- | --- | --- |
| ***qRT-PCR*** | | | |
| NtMYB3_Fw | | AGGAAATGCACAGAGGAGCT | MYB3-like /NCBI:XM_009598380.3 |
| NtMYB3_Rv | | GGTAAGTTACGCCAGCAACC |  |
| qRT_ Nt_DH29_F | | AAGATTTCCTGGAGCAAGCA | NtDH29/GB:JN390824.1 |
| qRT_ Nt_DH29_R | | TGGGGTAATGGCAAAATCAT |  |
| qRT_Nt_CV86_F | | CTCGGAATTCCCACAAAAGA | NtCV86/GB:JN390825.1 |
| qRT_ Nt_CV86_R | | CCTCTTAGCGACCAGTTTGC |  |
| qRT_Nt_AT1_F | | GCGTGGTTTTTCTCAACGAT | NtAT1/GB:JN390826.1 |
| qRT_Nt_AT1_R | | GCAAAGAAGGAGAGGGCTTT |  |
| qRT_NtSAMDC_F | | CACATTGGTGATGGAGTTGC | NtSAMDC/GB:AF321139.1 |
| qRT_NtSAMDC_R | | CGAACAACTCTTGTGCTGGA |  |
| **Cloning** | | | |
| FWprAN2C | GGGGACAAGTTTGTACAAAAAAGCAGGCTTCTTAGCTAGAATGCAACTCAGG | | 5' upstream sequence of ScAN2/scaffold4043 |
| RWprAN2C | GGGGACCACTTTGTACAAGAAAGCTGGGTCTTTTTTTTAATAATATATAGTTCTAAG | |  |
