# Supplementary material for: Expanded functional roles of R2R3-MYB (S6) transcription factors in balancing phenylpropanoid and phenolamide pathways in Solanaceae
Source: Plant Cell Physiol. 2025 Mar 13;66(6):878–89. doi: 10.1093/pcp/pcaf028 (PMC12290281; doi:10.1093/pcp/pcaf028)
Supplement: pcaf028_Supp [file pcaf028_supp.zip › suppl_data/pcp-2024-e-00254-File011.pdf]

Running titles: Phenolammides beyond anthocyanins

Supplementary data

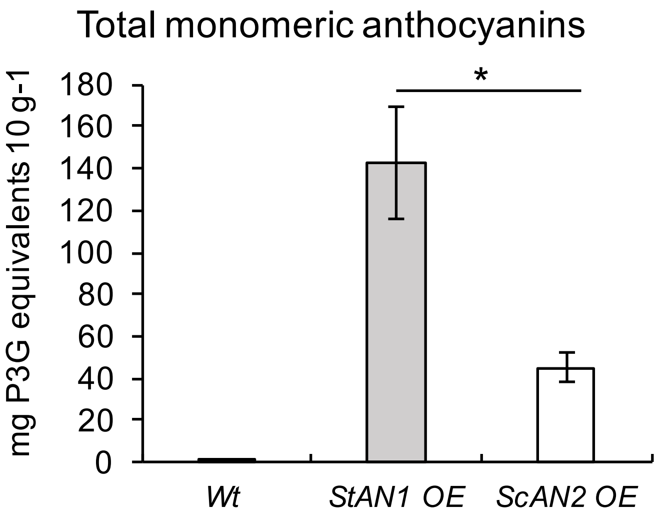

Supplementary Fig. S1. Total anthocyanin amount in fully expanded leaves of Wt, *StAN1* OE and *ScAN2* OE tobacco lines. Values are means±SD (n=3). Asterisks indicate statistically significant differences (P<0.05 value measured according to Student's t-test.).
