# Supplementary material for: Expanded functional roles of R2R3-MYB (S6) transcription factors in balancing phenylpropanoid and phenolamide pathways in Solanaceae
Source: Plant Cell Physiol. 2025 Mar 13;66(6):878–89. doi: 10.1093/pcp/pcaf028 (PMC12290281; doi:10.1093/pcp/pcaf028)
Supplement: pcaf028_Supp [file pcaf028_supp.zip › suppl_data/pcp-2024-e-00254-File014.pdf]

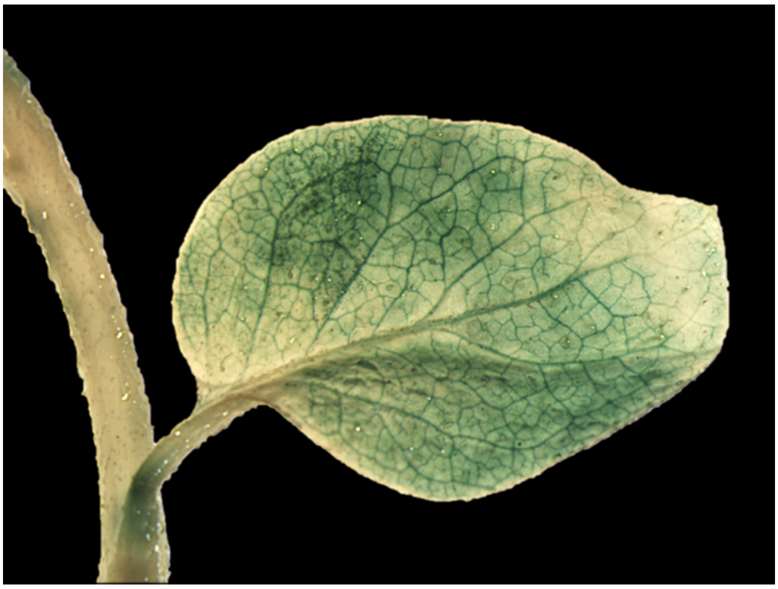

Supplementary Fig. S4. Macroscopic localization of GUS activity, in leaflets of potato plants under the control of pAN2 (≈1700bp) promoter.
