# Supplementary material for: Expanded functional roles of R2R3-MYB (S6) transcription factors in balancing phenylpropanoid and phenolamide pathways in Solanaceae
Source: Plant Cell Physiol. 2025 Mar 13;66(6):878–89. doi: 10.1093/pcp/pcaf028 (PMC12290281; doi:10.1093/pcp/pcaf028)
Supplement: pcaf028_Supp [file pcaf028_supp.zip › suppl_data/pcp-2024-e-00254-File015.pdf]

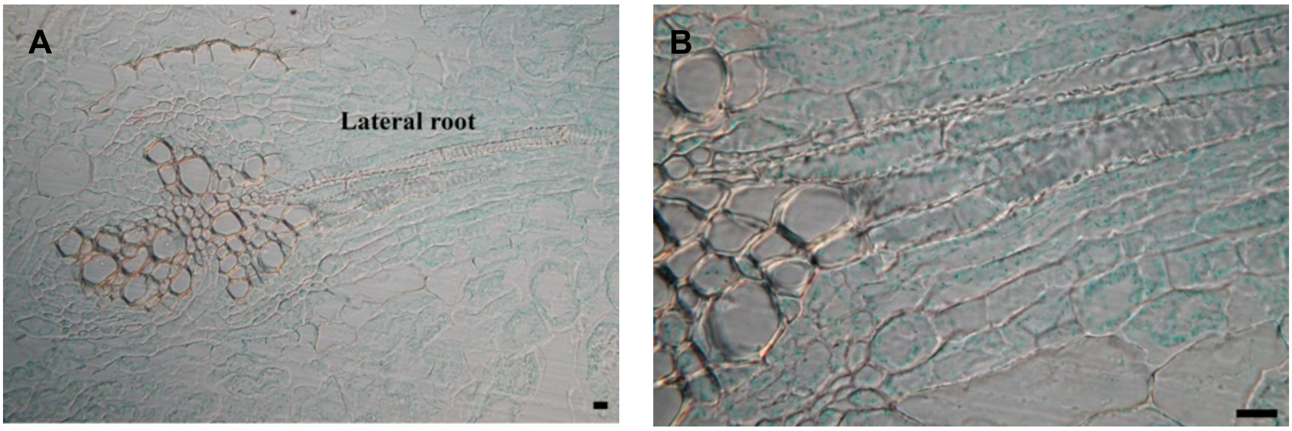

Supplementary Fig. S5. Microscopic localization of GUS activity, in roots of potato plant under the control of pAN2 (≈1700bp) promoter. In A and B, microscopic localization of lateral roots development.
